# How people explain their own and others’ behavior: a theory of lay causal explanations

**DOI:** 10.3389/fpsyg.2015.00139

**Published:** 2015-02-18

**Authors:** Gisela Böhm, Hans-Rüdiger Pfister

**Affiliations:** ^1^Bergen Laboratory for the Study of Decision, Intuition, Consciousness, and Emotion, Department of Psychosocial Science, Faculty of Psychology, University of Bergen, Bergen, Norway; ^2^Institute of Experimental Business Psychology (LueneLab), Leuphana University Lüneburg, Lüneburg, Germany

**Keywords:** social attribution, explanations, lay theories, causal inference, folk psychology

## Abstract

A theoretical model is proposed that specifies lay causal theories of behavior; and supporting experimental evidence is presented. The model’s basic assumption is that different types of behavior trigger different hypotheses concerning the types of causes that may have brought about the behavior. Seven categories are distinguished that are assumed to serve as both behavior types and explanation types: goals, dispositions, temporary states such as emotions, intentional actions, outcomes, events, and stimulus attributes. The model specifies inference rules that lay people use when explaining behavior (actions are caused by goals; goals are caused by higher order goals or temporary states; temporary states are caused by dispositions, stimulus attributes, or events; outcomes are caused by actions, temporary states, dispositions, stimulus attributes, or events; events are caused by dispositions or preceding events). Two experiments are reported. Experiment 1 showed that free-response explanations followed the assumed inference rules. Experiment 2 demonstrated that explanations which match the inference rules are generated faster and more frequently than non-matching explanations. Together, the findings support models that incorporate knowledge-based aspects into the process of causal explanation. The results are discussed with respect to their implications for different stages of this process, such as the activation of causal hypotheses and their subsequent selection, as well as with respect to social influences on this process.

## INTRODUCTION

People are usually not content with merely taking notice of others’ behavior; they ask why others behave the way they do and try to find explanations. Suppose, for example, that your next-door neighbor surprises you with a present. You will most probably find yourself wondering about why she does this. Is this simply a sign of her positive affection for you? Does she want to make a friend? Does she want to prepare you for an upcoming noisy party? Does she have a crush on you? The way you explain her behavior may affect not only your response to the gift, but also your attitude toward your neighbor and how your relationship will develop. The explanation of our own and others’ behavior is at the heart of social functioning. Explanations shape the way in which people make sense of the social world, how they perceive themselves and others as well as how they regulate their own behavior and react to others. The study of behavior explanations has traditionally been undertaken in attribution theory, where the explanation of a behavior has been conceived as consisting of the assignment of one or more causes to the behavior (e.g., Heider, 1958; Jones and Davis, 1965; Kelley, 1967, 1973). While other types of explanation exist (e.g., one can explain what a behavior is as in explaining a local custom to a foreigner; Antaki and Fielding, 1981), causal explanations are the most important type in that the vast majority of everyday explanations involve causality and the causal elements of an explanation are the parts that have the strongest influence on perceptions and judgments (Keil, 2006).

The present article focuses on the question of how people arrive at causal explanations of behavior and, more specifically, how such causal explanations are guided by lay theories. It is assumed that people hold implicit theories about what sorts of factors can be potential causes for a certain type of behavior; for example, that accomplishments can be brought about by high ability or strong effort. The aim is to specify these lay theories and identify which potential causes people consider when explaining everyday behavior. This aim constitutes a return to Heider’s (1958) seminal work that marks the origin of attribution research.

Heider’s approach contained two elements that have shaped the field of social attribution research (Heider, 1958; Hilton, 2007): First, he postulated his naïve analysis of action in which he specified the attributor’s prior causal knowledge and listed concrete causal factors that perceivers are assumed to use when explaining behavior (i.e., ability, effort, task difficulty, and luck). Second, he introduced mechanisms of causal inference such as covariation and discounting that guide the selection of one of these causal factors as an explanation for a particular behavior. Later work elaborated on the causal inference mechanisms, the most prominent approach being Kelley’s (1973) proposition of an intuitive analysis of variance as a specification of the covariation principle.

Heider’s idea of a lay causal theory of behavior has received less attention in subsequent attribution research. Interestingly, even though Heider (1958) introduced the notion of a lay psychology and this is generally seen as the foundation of attribution theory, work on lay theories seems more active in other areas of psychological research than in traditional attribution research. There is abundant research on lay theories as beliefs about some specific aspect of human experience (for a review see Levy et al., 2006), for example implicit theories about the malleability of personal attributes such as personality or intelligence (e.g., Plaks et al., 2005). Approaches that investigate lay causal theories as basis of causal explanations of behavior and try to specify concrete causal factors (such as ability or effort) that people consider relevant when explaining broad ranges of behavior, however, have been relatively scarce (for exceptions see Anderson, 1983a; Malle, 1999, 2004, 2011).

Approaches in the tradition of attribution theory that do address lay causal theories often look at them only with respect to the person-situation distinction (Hansen, 1980; Ybarra, 2002; Gawronski, 2004). That is, they only consider whether people hold hypotheses about personal or situational causation; they do not distinguish more specific causal factors within the person or situation, such as ability or effort as factors within the person. However, recent research has shown that when people try to understand behavior, they do not so much ask whether it was due to the person or the situation but rather which specific intention, goal, motive, or disposition led to the behavior (Malle, 1999, 2004; Malle et al., 2000; Kammrath et al., 2005). For example, Reeder et al. (2004) found that the attribution of a disposition to an actor could be better predicted from the specific motives that are ascribed to the actor (e.g., whether she is seen as pursuing obedient or selfish goals) than from global attributions of the behavior to the person or the situation. Moreover, the content of specific motives and dispositions that are attributed to a target person has been found to be crucial for subsequent judgments and behavioral predictions (Reeder et al., 2004; Kammrath et al., 2005). These findings suggest that causal explanations are more specific than a mere attribution of the behavior to the person or situation, and that the elements of lay theories of behavior consist of more concrete causal factors such as goals or dispositions.

Some authors investigated the role of general knowledge structures in causal attribution (Lalljee and Abelson, 1983; Hilton and Slugoski, 1986; Read, 1987; Abelson and Lalljee, 1988). Most of them draw on Schank and Abelson’s (1977) theory and argue that people use knowledge structures such as scripts, plans, goals, and themes when explaining behavior. According to these models, perceivers assimilate an observed behavior to their knowledge structures and derive an explanation of it from their general world knowledge. They might, for example, explain a behavior by the plan that the actor is likely to pursue. These knowledge-based models provide a general conceptual framework of information processing rather than a specific model of causal explanations. The theoretical framework proposed in the present article draws on these models but tries to specify structures of prior knowledge that are more specific to the process of causal explanation.

In the present article, a model is presented that emphasizes the importance of preconceptions about causal relationships in guiding explanations of behavior, the *causal explanation network (CEN)*. The basic assumption of the CEN model is that the first step in understanding is to classify an observed behavior as a certain type of behavior, and that different types of behavior trigger different hypotheses about the causes of the behavior. Consider, for instance, a tennis player who wins a match. What are possible causal explanations for this victory? The player may conclude, for example, that she is a naturally gifted tennis player, or that she practiced hard to be in good shape. In this example, the victory is classified as an achievement outcome and possible explanations are, for example, ability (natural talent in this example) or effort (hard practice). Effort is not a plausible explanation for other types of behavior, for example for someone’s being sad, because we do not normally expect anybody to make an effort in order to be sad. Rather, we would search for an external event that could have caused the person’s sadness.

Hence, depending on the type of behavior that is to be explained, different causal hypotheses come to mind. The CEN model specifies these causal hypotheses. In the next section, we outline the CEN model. Two experiments are reported that tested predictions derived from the CEN model. The first experiment investigated which types of behavior people differentiate and whether causal explanations are generated according to the rules that are specified in the CEN model (described below). The second experiment tested the hypothesis that causal explanations that adhere to the model are generated faster and more frequently than explanation types that violate the rules specified in the CEN model.

## THE CAUSAL EXPLANATION NETWORK

The assumption that different types of behavior elicit different causal explanations has been an early hypothesis in attribution research. The perceived intentionality and controllability of behaviors turned out to be important criteria for distinguishing behavior types (Malle, 1999, 2004, 2011). For example, it has been shown that personal reactions such as emotions (e.g., “Mary is afraid of the dog”) or opinions (e.g., “Bill thinks his teacher is unfair”) are perceived as reactions to a stimulus, whereas actions (e.g., “Peter cheats on the exam”) and achievements (e.g., “Sue is admitted to Harvard”) are seen as being brought about by the actor (Heider, 1958; McArthur, 1972; Hansen, 1980; Hilton, 2007). Hilton (2007) even argues that the distinction between emotions and actions is innate in that their perception is based on distinct, innate and hard-wired modules in the human brain.

A related distinction that also emphasizes intentionality and controllability as important criteria for distinguishing behavior types is that between reasons and causes. This distinction has a long history in philosophy; relevant for current purposes are only those approaches that discuss reasons and causes as different types of lay explanations (Buss, 1978, 1979; for critical discussions see Harvey and Tucker, 1979; Kruglanski, 1979; Locke and Pennington, 1982). Reason explanations are explanations of intentional behavior that refer to the agent’s reasons for acting that way, for example “Jim entered the store because he wanted to buy a book.” Cause explanations, in contrast, are explanations of unintentional behavior that refer to the causes that brought about the behavior without an intervening intention, for example “Sue broke her leg because she tripped over a tree root” (Malle, 1999).

As the basic assumption of the CEN model is that different types of behavior elicit different types of explanations, one of its constituents is a classification of behaviors and explanations. The categories of such a classification should be sufficiently universal and encompassing to be applied to the explanation of behavioral sequences. People often ask themselves not only why a particular behavior occurred, but also what happened before and what may happen next. We propose a unified taxonomy to classify both behaviors and explanations rather than two separate taxonomies. Thus, in CEN the same set of categories serves to classify behaviors as well as explanations.

The categories were chosen in such a way that they differ with respect to the behavior’s perceived intentionality, and with respect to the attributional dimensions that are traditionally assumed in the literature (e.g., Heider, 1958; Weiner, 1985, 1986): the locus (internal–external), stability, and controllability of the cause. We assume that categories that differ on these dimensions are particularly useful in serving the functions of causal explanations; the functions that are usually mentioned in the attribution literature are understanding, predicting, and controlling behaviors and events as well as protecting the self and social identity, for example through self-esteem enhancement and positive self-presentation to others (Forsyth, 1980).

**FIGURE 1:**
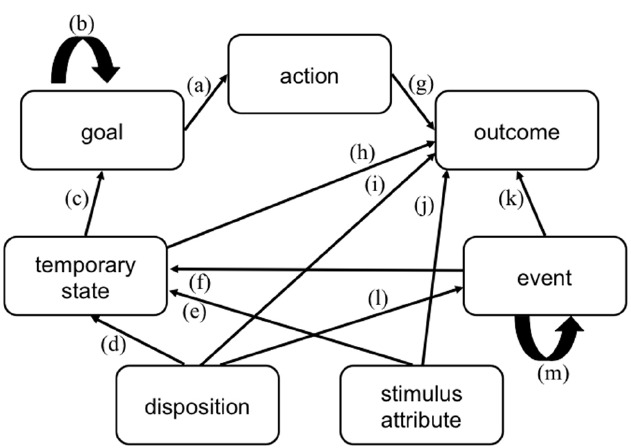
**The causal explanation network (CEN) model.** Arrows indicate which factors are assumed to be causally linked in the lay theory of behavior. The letters refer to the inference rules specified in the text.

The causal explanation network (CEN, see Figure 1) specifies seven categories that are assumed to be relevant in lay causal thinking about an actor’s behavior: (i) the actor’s *goals*, that is, future states that the actor strives for; (ii) his/her enduring *dispositions*, such as personality traits, attitudes, or skills; (iii) his/her *temporary states*, such as emotions, evaluations, mental states, motivational states, or bodily states; (iv) his/her *actions*, that is, behaviors that are perceived as intentional and goal-directed; (v) his/her action *outcomes*, that is, whether the aim of an action is fulfilled or not, typical outcomes are achievement outcomes; (vi) uncontrollable *events*, that is, events that happen to a person and that were not intended, such as accidents; and (vii) *stimulus attributes*, that is, features of the person or object toward which a behavior is directed, such as the difficulty of an exam that the actor wants to pass or the beauty of a picture that the actor admires.

As described earlier, these categories are used to classify behavior as well as to generate causal explanations. Furthermore, it is assumed that these categories are mentally represented as causally linked to each other and that people implicitly apply the following inference rules when explaining behavior (as indicated by the arrows in Figure 1, examples are given below): (a) actions are caused by goals, (b) goals are caused by (higher order) goals, (c) goals are caused by temporary states, (d) temporary states are caused by dispositions, (e) temporary states are caused by stimulus attributes, (f) temporary states are caused by events, (g) outcomes are caused by actions, (h) outcomes are caused by temporary states, (i) outcomes are caused by dispositions, (j) outcomes are caused by stimulus attributes, (k) outcomes are caused by events, (l) events are caused by dispositions, (m) events are caused by (preceding) events.

The categories and inference rules have been derived from the body of attribution research. For example, the causes that Heider (1958) specifies in his naïve analysis of action are particularly suited as explanations for achievement outcomes and are thus reflected in the arrows that are directed toward outcomes in CEN: actions (which correspond to effort in Heider’s terms), dispositions (ability), stimulus attributes (task difficulty), and events (luck). Lay theories of intentional actions have been analyzed in the knowledge-based approaches to attribution which were mentioned earlier (Lalljee and Abelson, 1983; Leddo et al., 1984; Leddo and Abelson, 1986; Read, 1987; Abelson and Lalljee, 1988). This idea has been refined by Malle (1999, 2004, 2011) and Malle and Knobe (1997). They find that the most important components of a lay theory—or folk concept as they call it—of intentionality are the desire for an outcome and the belief that the behavior leads to the outcome, both are seen as giving rise to an intention to act. In terms of the CEN categories, beliefs refer to temporary (mental) states, and desire for an outcome refers to a goal. There is general agreement in the literature that people see intentions (which correspond to goals in CEN) as immediate causes of actions (e.g., Heider, 1958; Jones and Davis, 1965; Malle, 1999, 2011). Furthermore, research on dispositional inference has shown that dispositions are inferred via inferences about the target person’s intentions and motives, which correspond to goals in CEN (Jones and Davis, 1965; Reeder et al., 2004; Kammrath et al., 2005). Early work has shown that temporary states (emotions, opinions) are attributed to the stimulus (Heider, 1958; McArthur, 1972).

The categories and inference rules in CEN are based on these existing attribution theories and empirical findings. The aim of CEN is to provide a unifying framework that can integrate these diverse approaches and is applicable to a broader range of attribution situations than most models. While the basic idea that different behavior types trigger different explanations has been proposed before, the specific set of categories and inference rules postulated in CEN is new. Furthermore, to our knowledge no other attribution model has postulated the same set of categories for classifying both behavior types and explanation types. One great advantage of this proposition is that CEN can—with a parsimonious set of assumptions—describe how people explain extended behavioral sequences and construct behavioral episodes (e.g., John is a nice guy; when he saw the old lady with her heavy bag, he felt sorry for her and wanted to help. Thus, he carried her bag to her home.).

## THE PROCESS OF EXPLANATION

According to CEN, the explanation process begins with classifying the behavior that is to be explained as a member of one of these categories. The model predicts that a behavior is attributed to those explanation types that are cognitively represented as being causally linked to the behavior category. The same category can serve as a behavior type in one case and as an explanation type in another case. For example, according to inference rule (a) intentional actions are caused by goals. According to this rule, behavior that is perceived as an *intentional action* is explained by the action’s goal. When asking why a person strives for a certain *goal*, in turn, the model predicts a higher order goal (rule b) or a temporary—for example, emotional—state (rule c) as an explanation. The action “John calls a friend,” for example, may be explained by his goal “he wants to invite his friend.” This goal could be explained by a higher order goal (e.g., “John wants to be social”), or by a temporary state (e.g., “John is bored”). An *outcome* (e.g., “John fails his exam”) may be explained by an action (e.g., “he did not study hard enough,” rule g), by a temporary state (e.g., “he was nervous,” rule h), by a disposition (e.g., “he is a dull person,” rule i), by a stimulus attribute (e.g., “the exam was too difficult,” rule j), or by an event (e.g., “bad luck,” rule k). *Uncontrollable events* (e.g., “John’s basement is flooded”) may be explained by dispositions (e.g., “John is an unlucky fellow,” rule l), or by preceding events (e.g., “a water pipe broke,” rule m).

As these examples suggest, the inference rules are assumed to be transitive so that the explanation of extended behavior sequences can be incorporated in the model. There are no causes assigned to dispositions and stimulus attributes. Dispositions and stimulus attributes are assumed to mark the end of a causal search, to be particularly satisfactory explanations, and to serve as ultimate explanations that do not raise any further questions. The outcome-category is the one that has no consequences, because outcomes mark the end of a behavioral sequence.

These inference rules are assumed to reflect lay conceptions about what sorts of things may cause what other sorts of things. They may be thought of as a cognitively represented causal syntax. The question of whether these rules reflect “true” causal relationships is not relevant for a lay theory. The aim is to describe a lay theory which people use in their everyday lives when they give subjective and intuitive explanations. Taken as a scientific theory about human behavior, such folk theories might be completely wrong. The CEN rules were derived from other attribution models (e.g., Heider, 1958; Jones and Davis, 1965; Lalljee and Abelson, 1983; Read, 1987; Abelson and Lalljee, 1988; Malle, 1999), and are assumed to reflect common sense. Thus, they are the rules that are empirically expected to be employed by many people. Two experiments will be reported that test predictions derived from the CEN model.

## EXPERIMENT 1: THE STRUCTURE OF BEHAVIORS AND FREE-RESPONSE EXPLANATIONS

Experiment 1 aimed at investigating three questions. The first two questions address the appropriateness of the seven categories as a description of the cognitive structure of behaviors and explanations. The CEN model assumes that these seven categories are used when encoding behavior as well as when generating explanations.

The first research question of Experiment 1 is whether people actually classify behavioral episodes in a way that corresponds to the CEN categories. Participants were presented with descriptions of behavioral episodes, which had been constructed in such a way that *a priori* each behavior belonged to one of the categories. Participants were asked to judge the similarity between these behaviors. Similarity judgments are a non-directive type of judgment that leaves it to the judges which attributes of the stimuli they use to evaluate similarity. The assumption is that judges base their judgment on those attributes that are most salient or important to them. Therefore, similarity judgments are often used in cognitive psychology as an indirect and unobtrusive measure of cognitive relationships (Tversky, 1977; Nosofsky, 1992). A multidimensional scaling analysis of the similarity judgments was expected to yield a configuration of the behaviors that corresponds to their *a priori* assignment to the CEN categories. A secondary aspect of the first research question is whether the presented behavior types differ in their perceived intentionality and controllability, which are assumed to be distinguishing dimensions of the behaviors.

The second research question is whether the CEN categories sufficiently capture the types of explanations that people give. That is, can explanations that are generated in an unrestricted way be allocated to the seven categories or are there any important categories lacking that would be needed to classify explanations? In order to answer this question, explanations were obtained in a free-response format and coded by independent raters according to the seven categories.

The third research question of Experiment 1 refers to the core prediction in the CEN model, namely, that explanations are generated according to the postulated inference rules. That is, when people generate an explanation, does the type of this explanation correspond to a category that the CEN model assumes to be linked to the category of the behavior? For instance, are actions explained by goals and outcomes by actions, states, or stimulus attributes? This question was addressed by analyzing the relationship between the type of the presented behavior and the explanation type of the free-response explanations.

An additional aim of this study is to further test the validity of the CEN model by replicating two attributional tendencies that have been reported in the attribution literature. One such finding is that actors and observers differ in their attributions in such a way that actors tend to focus on situational factors when explaining their behavior whereas observers tend to see others’ behavior as caused by dispositions. This so called actor–observer difference has been introduced by Jones and Nisbett (1971); it has entered the textbooks and has been described as a stable, robust, and well-established phenomenon (for example in a meta-analysis by Watson, 1982). A more recent meta-analysis casts some doubt on the pervasiveness and robustness of the actor–observer difference: Malle (2006) found that many studies failed to replicate the actor–observer effect and that average effect sizes vary around 0. Nevertheless, this meta-analysis also showed that one of the conditions under which differences between actors and observers occur is when free-response explanations were obtained, as in the present study. Therefore, the present study investigated whether the actor–observer divergence can be established when explanations are analyzed by means of the CEN categories.

Another attributional tendency is that positive outcomes are attributed to internal, dispositional factors, whereas negative outcomes are attributed to situational causes (e.g., Davis and Stephan, 1980; McAllister, 1996; Sedikides et al., 1998; for reviews see Miller and Ross, 1975; Zuckerman, 1979). This *self-serving* or *positivity bias* has proved as a stable and general phenomenon in attribution research that has been found in different behavioral domains, and for own as well as for others’ outcomes (Böhm, 1992; Duval and Silvia, 2002; Moon, 2003; for meta-analyses that focus on the self-serving bias, i.e., on favorable attributions with respect to the self, see Mezulis et al., 2004, as well as Campbell and Sedikides, 1999).

The present study aimed to replicate these two attributional tendencies by varying the perspective of the attributor as actor or observer on the one hand, and the valence of the behavior as positive or negative on the other hand. With respect to the attributor’s *perspective*, observers were expected to give more dispositional explanations than actors, whereas actors were expected to give more explanations than observers that refer to the three external categories: outcomes, events, and stimulus attributes. With respect to the behavior’s *valence*, potential differences between actors and observers were, for the sake of simplicity, not considered. A positivity bias was expected for both perspectives in such a way that internal explanations are preferred for positive behaviors and external explanations for negative behaviors. In terms of the CEN categories, this means that explanations referring to goals, actions, states, and dispositions were expected to be more frequent for positive than for negative behaviors, whereas explanations referring to outcomes, events, and stimulus attributes were expected to be preferred for negative behaviors.

### METHOD

Both experiments reported in this article conformed at all stages with the ethical principles of the German Research Council (DFG); informed consent was obtained from all participants.

#### Participants

Fifty undergraduate psychology students volunteered to participate. They received course credit for their participation. Their age ranged from 19 to 47 years (*M* = 24.9); 78% were female.

#### Behavior descriptions

Twenty behavior descriptions were used. The behavior descriptions corresponded to four of the seven categories: actions, outcomes, states, and events. Goals, dispositions and stimulus attributes were not presented as behavior descriptions, because they do not constitute observable behaviors. States were further distinguished in emotions and evaluations (cf. McArthur, 1972). Hence, five behavior types were presented: action, outcome, state-evaluation, state-emotion, and event (behavior type manipulation). For each type, two desirable and two undesirable behaviors were presented (valence manipulation). Furthermore, the behavior descriptions were formulated either in the first or in the third person (perspective manipulation). Half of the participants received all behavior descriptions in the first person (actor perspective), the other half of the participants in the third person (observer perspective^1^). The items read as follows: (a) action-positive: “<I carry/Matthew carries> an old lady’s bag to her home,” “<I donate/Cloe donates> a large sum of money to Greenpeace”; (b) action-negative: “<I baste/Michael bastes> a passer-by,” “<I tell my friend/Ruth tells her friend> a lie”; (c) outcome-positive: “<I pass/Andy passes> the intermediate exam,” “<I get the job for which I/Warren gets the job for which he> applied”; (d) outcome-negative: “<I do not get the apartment that I/Kathy does not get the apartment that she> wanted,” “<I fail/Harry fails> the drivers test”; (e) state-evaluation-positive: “<I like my/Julie likes her> new bike,” “<I like my friend’s/Nick likes his friend’s> new hair cut”; (f) state-evaluation-negative: “<I find my friend’s/Monica finds her friend’s> cloths tasteless,” “<I think my/Ben thinks his> teacher is unfair”; (g) state-emotion-positive: “<I am very happy with my/Sheila is very happy with her> holiday flirt,” “<I am pleased with my/Becky is pleased with her> birthday gift”; (h) state-emotion-negative: “<I am/Sue is> angry at the car driver,” “<I am/Mary is> afraid of the dog”; (i) event-positive: “<I receive/Tom receives> a trip to Hawaii as a gift,” “<I win/Steve wins> in a lottery”; (j) event-negative: “<My/Chuck’s> basement is flooded,” “<My/Sally’s> wallet was stolen.”

#### Design and procedure

Three independent variables were manipulated by providing the behavior descriptions: (a) behavior type with five levels (action, outcome, state-evaluation, state-emotion, event); (b) the valence of the behavior (positive vs. negative), and (c) the perspective of the attributor (actor vs. observer). Behavior type and valence were manipulated within, perspective between subjects.

The experiment was run in groups of three to four participants who worked independently on their tasks. Upon arrival, they were instructed that they were to read descriptions of a number of situations and that they should imagine either that they were in that situation themselves (actor perspective) or that they observed someone who was in that situation (observer perspective). They were randomly assigned to the two perspective conditions.

#### Dependent measures

Each participant received the 20 behavior descriptions three times, each time completing one of three judgmental tasks. The order of the behavior descriptions was randomized for each participant and each judgmental task. Participants completed the following three judgmental tasks.

First, free-response explanations for the behavior descriptions were obtained. Participants received a booklet in which each behavior description was written on top of a separate page. Participants were asked to explain in free-response format why they thought the behavior probably occurred. Participants were asked to answer briefly and spontaneously and to generate exactly one explanation for each behavior item.

Second, participants judged the similarity of the behavior descriptions. The behavior descriptions were each printed on a separate card. Participants were asked to sort the behavior descriptions into piles of similar behaviors.

Third, three evaluative ratings were obtained in a questionnaire. Participants evaluated the intentionality, controllability, and desirability of each behavior. Each rating was given on a rating scale ranging from 0 (*not at all*) to 4 (*very much*).

Additional measures were obtained that are beyond the scope of this paper and will not be reported here. They relate to the following constructs: importance of explanation types, information search, behavioral predictions. None of these measures yields results that would lead to different conclusions than are drawn here.

#### Coding of free-response explanations

The free-response explanations were allocated to explanation type categories by independent raters. That is, raters judged for each explanation whether it referred to a goal, an action, an outcome, an event, a state, a disposition, or a stimulus attribute, or if it did not fit in any of these categories. For instance, an explanation such as “Matthew carries the old lady’s bag, because he wants to help her” was coded as a goal, an explanation such as “Matthew is a nice/helpful person” was coded as a disposition. Three raters coded all explanations that were given from participants in the actor condition, and another three raters coded the explanations from observer-condition participants. Thus, each rater coded 500 explanations (25 participants in each perspective condition gave one explanation for each of 20 behavior descriptions). The interrater agreement between the three raters of a condition as measured by Fleiss’ Kappa is κ = 0.62 for the three raters in the actor condition and κ = 0.63 for the three raters in the observer condition. According to Landis and Koch (1977) these values can be considered to reflect very good agreement. An explanation was assigned to an explanation type category if at least two of the three raters agreed on that category. Explanations that were assigned to different categories by all three raters were not used in further analyses; in this way, 91.3% of the explanations were assigned to an explanation type.

#### Construction of a similarity matrix

The similarity sortings were used to construct a pairwise similarity matrix of the behavior descriptions. For each pair of behavior items, the number of participants who had sorted that pair into the same pile was used as a measure of the similarity of that pair.

### RESULTS

#### Evaluative ratings

The evaluative ratings served as manipulation checks. The *a priori* behavior types differ significantly with respect to their perceived intentionality, *F*(4,45) = 118.5, *p* < 0.001, η^2^ = 0.913. The pattern of means is in the expected direction, the judged intentionality of the behavior decreases from actions (*M* = 2.24) via outcomes (*M* = 2.01), state-evaluations (*M* = 1.02), and state-emotions (*M* = 0.74) to events (*M* = 0.59).

The behavior types also show the expected effect on perceived controllability, *F*(4,45) = 169.86, *p* < 0.001, η^2^ = 0.938. The pattern of the means is similar to that for intentionality. The judged controllability decreases from actions (*M* = 3.55) via outcomes (*M* = 2.60), state-emotions (*M* = 1.77), and state-evaluations (*M* = 1.73) to events (*M* = 1.18).

Positive and negative behaviors significantly differ in their perceived desirability, *F*(1,48) = 1090.59, *p* < 0.001, η^2^ = 0.958. Positive behaviors are judged as much more desirable (*M* = 3.58) than negative behaviors (*M* = 0.36). Thus, the valence manipulation was successful.

In sum, the analysis of the evaluative ratings renders two main results. First, the behavior types systematically differ with respect to their intentionality and controllability. The order of the behavior types is as hypothesized. Actions are perceived as the most intentional and controllable, followed by outcomes and temporary states (evaluations and emotions); events are the least intentional and controllable behavior types. Second, valence of the behaviors was successfully manipulated.

#### The structure of behaviors

**FIGURE 2:**
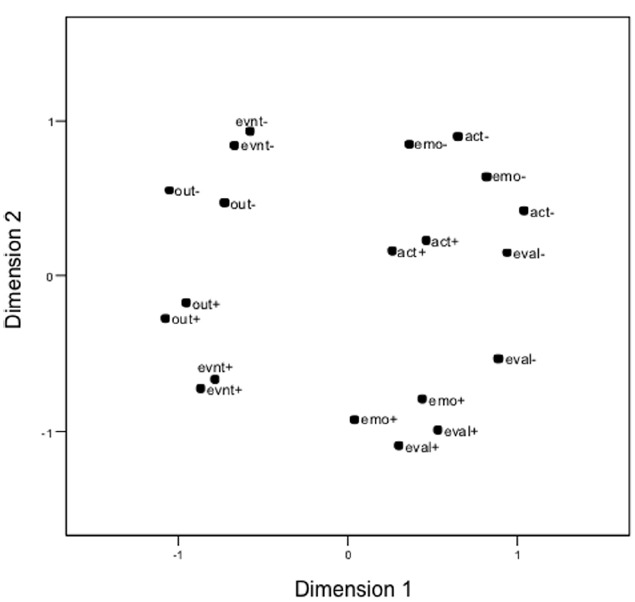
**Multidimensional scaling solution of behaviors based on similarity sortings, Experiment 1.** act, action; emo, emotion; eval, evaluation; out, outcome; evnt, event; +, positive behavior; –, negative behavior.

In order to analyze the perceived structure of the behaviors, the similarity matrix that was derived from the sorting task was subjected as input to a non-metric multidimensional scaling analysis (Borg and Groenen, 2005). The actor and observer conditions yield identical configurations (the pairwise distances of the behaviors in the two resulting configurations correlate with *r* = 0.90). Therefore, the configuration for the entire sample will be reported. The two-dimensional solution yields a good fit, *stress* = 0.175 (non-metric MDS, stress formula 1); it is shown in Figure 2. A third dimension (fit for the three-dimensional solution is *stress* = 0.06) provides some differentiations between the behaviors that will be pointed out below.

The distance of the behaviors in the configuration reflects their similarity. That is, the closer two behaviors are located the more participants decided that these two behaviors are similar and placed them into the same pile. The configuration shows that the two behaviors that are of the same type and valence are grouped closely together and are thus perceived as very similar. The negative emotions are interspersed with negative actions in this figure, but they are apart on the third dimension that is not displayed. The horizontal axis separates the situational factors (outcomes, events) that are located on the left from the personal factors (actions, emotions, evaluations) on the right. The vertical axis separates positive behaviors at the bottom from negative behaviors at the top. Interestingly, negative emotions are located close to actions, to negative as well as to positive actions, whereas positive emotions are close to positive evaluations and relatively far apart from actions. This seems to imply that negative emotions are much more strongly associated with behavioral impulses, thus actions, than positive emotions.

This multidimensional scaling analysis supports the CEN categories as a cognitive taxonomy of behaviors. The behaviors that are assumed to belong to one category in CEN were perceived as similar by participants, which supports the assumption that participants perceived them as members of the same category. The cognitive structure of the behaviors is also influenced by the valence of the behaviors. This might be expected, as the perceived intentionality is assumed to be a basis for the cognitive structuring of behaviors and positive behaviors generally appear more intended than negative behaviors.

#### Free-response explanations

The Behavior Type × Explanation Type contingency table (Table 1) shows that 91.3% of the open-ended explanations could be classified according to the CEN explanation types. Thus, the CEN categories cover a large proportion of the explanation types generated in free-response explanations by participants.

**TABLE 1:** **Number of free-response explanations by explanation type and behavior type (Experiment 1)**.

**Explanation type**								
**Behavior type**	**Goal**	**Action**	**Outcome**	**Event**	**Temporary state**	**Disposition**	**Stimulus attribute**	**Sum**
Action	28	3	2	19	60	38	34	184
Outcome	2	12	48	34	21	47	14	178
Evaluation	3	1	9	10	20	40	102	185
Emotion	6	0	5	42	30	12	80	175
Event	0	12	24	98	27	9	21	191
Sum	39	28	88	203	158	146	251	913

A total of N = 1000 free-response explanations were generated across all behavior descriptions and all participants.

**FIGURE 3:**
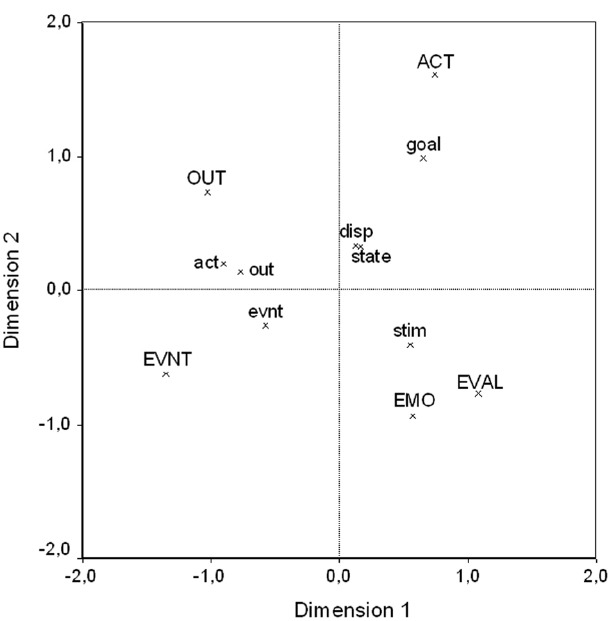
**Asymmetric correspondence analysis plot of free-response explanations, Experiment 1.** Behavior types are in standard coordinates, explanation types in principal coordinates. Percentage of inertia: Dimension 1 = 50.43%, Dimension 2 = 26.65%. Behavior Types are depicted in upper case letters, explanation types in lower case letters. ACT, act: action; EVAL: state-evaluation; EMO: state-emotion; OUT, out: outcome; EVNT, evnt: event; goal: goal; state: temporary state; disp: disposition; stim: stimulus attribute.

The contingency table also shows that the type of the generated explanation depended on the type of the behavior that was explained, χ^2^(24) = 482.79, *p* < 0.001. The relationship between behavior type and explanation type was further analyzed by asymmetric correspondence analysis (Greenacre, 1984, 1993). This procedure is similar to principal components analysis and provides a graphical representation of the relationship between the rows and columns of a contingency table. The two-dimensional solution accounts for 77.1% of the variance; it is depicted in Figure 3. Behavior types (upper case labels in Figure 3) are represented in standard coordinates, explanation types (lower case labels) in principal coordinates (Greenacre, 1984). The plot shows that participants to a large extent obeyed the postulated inference rules. That is, they preferably generated those explanation types that are assumed to be causally linked to the behavior category according to the CEN model. Each quadrant depicts a typical relationship. Actions were mainly explained by goals, and somewhat less typically by dispositions and temporary states.^2^ Evaluations and emotions were mainly explained by stimulus attributes, events by other events, and outcomes by prior actions and outcomes.

One aspect of these findings is noteworthy. The CEN model assumes a direct link to actions only from goals. Participants explained actions not only by goals, but also by temporary states and dispositions of the actor. This finding does not contradict CEN. According to CEN, states and dispositions are indirectly linked to actions as remote causes; thus, they may well serve as explanations for actions. The finding is noteworthy, because it indicates that people give proximate as well as distal causes as explanations. The role of such causal chains in explanations will be addressed in the second experiment.

There is only one deviation of the findings from the CEN model: Behavioral outcomes were explained by previous outcomes. When formulating the CEN model, it was assumed that outcomes mark the end of a behavioral episode and would therefore constitute some terminal point in explanatory activities. Participants, however, apparently constructed sequences of such behavioral episodes when explaining outcomes. For instance, when explaining why Warren gets the job for which he applied, they gave explanations such as “because he received good grades,” or “because he made a good impression during the interview.” Apparently, achievement outcomes give rise to future achievement outcomes in lay reasoning.

#### Attributional biases

Table 2 shows the distributions of the free-response explanations across the explanation types for the actor–observer difference (upper half of Table 2) and for the positivity bias (lower half).

**TABLE 2:** **Actor–observer difference (top) and positivity bias (bottom) in free-response explanations (Experiment 1): distribution of free-response explanations across explanation types**.

Explanation type								
	**Goal**	**Action**	**Outcome**	**Event**	**Temporary state**	**Disposition**	**Stimulus attribute**	**Sum**
Actor–observer differences								
Actors	26	11	37	102	71	47	164	458
Observers	13	17	51	101	87	99	87	455
								
Positivity bias								
Positive behaviors	23	23	57	80	54	87	126	450
Negative behaviors	16	5	31	123	104	59	125	463

The attributor’s perspective affected the type of explanation that was generated, χ^2^(6) = 51.6, *p* < 0.001. This effect was particularly pronounced for the two explanation types disposition and stimulus attribute: Observers gave more dispositional explanations than actors, whereas the reverse holds for explanations that refer to stimulus attributes. These two differences were expected. However, actors and observers were also expected to differ with respect to the other two situational explanations (i.e., outcomes and events). The results show that actors and observers did not differ in all sorts of situational explanations, but only in specific types of situational attributions. These results confirm the actor–observer hypothesis as it was originally formulated by Jones and Nisbett (1971). They claimed that observers tend to attribute behavior to the actor’s stable dispositions, whereas actors are inclined to see their behavior as caused by situational requirements. Jones and Nisbett (1971) already hypothesized that actors and observers do not generally differ with respect to personal and situational attribution, but with respect to specific internal causes (i.e., dispositions) and specific external causes (i.e., situational requirements). Possibly, stimulus attributes are the type of external factor that qualifies as a situational requirement, more so than uncontrollable events and outcomes.

Table 2 also shows that positive and negative behaviors elicited different types of explanation, χ^2^(6) = 50.6, *p* < 0.001. Goals, actions, outcomes, and dispositions were more frequent explanations for positive than for negative behaviors. Events and temporary states, in contrast, were much more frequently given as explanations for negative than for positive behaviors. There was no difference in the number of stimulus-attribute explanations generated for positive and negative behaviors.

Many studies on the positivity bias have focused on the attribution of success and failure and have investigated attributions only with regard to the internal–external dimension (Campbell and Sedikides, 1999; Mezulis et al., 2004). Again, the CEN explanation types provide a more differentiated picture. It does not hold true that positive behaviors are generally attributed to internal and negative behaviors to external causes. Most of the internal explanation types (goal, action, disposition) are preferred for positive behaviors, but temporary states as internal attributions are preferred for negative behaviors. And events are the only external explanation type that is preferred for negative behaviors; outcomes are more frequent for positive behaviors, and stimulus attributes are equally often named as explanations for positive and negative behaviors. Uncontrollable events and temporary states were preferred to explain negative behaviors. These two explanation types seem particularly suited to avoid blame, which supports a motivational interpretation of the positivity bias (Zuckerman, 1979). Hence, controllability and stability may be as important aspects of the positivity bias as the locus of the attribution on the internal–external dimension. Grove et al. (1991) even found that success and failure attributions differed only with respect to stability and controllability, and not at all with respect to their locus. Hence, the study of the stability and controllability dimensions may deserve more attention than they have so far received in the positivity bias literature (for a similar argument see Mezulis et al., 2004).

### DISCUSSION

This experiment demonstrates that the seven categories postulated in the CEN model cover the cognitive concepts that are relevant in the lay theory of behavior. This is demonstrated by two aspects of the findings. First, when asked to sort behaviors according to their similarity, participants sorted them according to their theoretically assumed behavior types. Hence, the CEN categories seem to capture the perceived similarity of behaviors. Remember that similarity judgments are non-directive. We gave no hint as to which attributes of the behaviors should be used to judge their similarity. Apparently, the CEN categories provide a structure that corresponds to the mental representation of behaviors and that is used spontaneously when evaluating the similarity of behaviors in an explanatory context. It may be argued that the presented behaviors were constructed so that they corresponded to the CEN categories, which may have restricted the diversity of the behaviors and thus the range of attributes that participants could use as a basis for their similarity judgments. However, apart from the general problem that any selection of behaviors implies some restriction of range, we believe that the behaviors still differed on many other attributes than the ones conforming to the CEN model (e.g., sex of protagonist in the observer condition) so that the result that participants’ similarity sortings corresponded to the CEN categories can be seen as supportive evidence for the CEN model. Second, most of the explanations generated in free-response format correspond to one of the postulated explanation types. These types obviously cover the concepts that people use when thinking about explanations. Taken together, these two results support the assumption that the CEN categories are used for encoding behavior as well as for generating explanations.

The findings support not only the assumption that the seven concepts are used in explanatory activities, but also that the search for causes proceeds along the inference rules that are assumed in the CEN model. The free-response explanations that were generated by participants to a large degree conformed to the explanation types that the CEN model predicts for a given behavior type: Actions were explained by goals, temporary states, and dispositions; outcomes were explained by preceding actions; temporary states, namely evaluations and emotions, were explained by stimulus attributes; and events were explained by antecedent events. The only unexpected result is that outcomes were frequently explained by previous outcomes.

There is also evidence for two attributional biases, namely actor–observer differences and positivity bias. With respect to the former, results show that actors prefer dispositional explanations, whereas observers prefer explanations that refer to stimulus attributes. With respect to the positivity bias, negative behaviors are by far more frequently attributed to events and temporary states than are positive behaviors. Goals, actions, outcomes, and dispositions, in contrast, are more frequently cited as explanations for positive than for negative behaviors. Thus, the findings replicate these two biases, which supports the validity of the CEN categories as explanation types. Furthermore, since the CEN categories classify explanations on a more detailed level than most other attribution models, they allow us to consider these attributional biases on a more differentiated level. For the actor–observer difference, it is not situational factors in general but stimulus attributes in particular that are preferred by actors. Similar results were found by Malle et al. (2007) who also studied the actor–observer asymmetry my means of free-response explanations. These explanations were coded in two ways: On the one hand with respect to the global person-situation dichotomy and on the other hand concerning differentiated factors such as the actor’s reasons (i.e., beliefs and desires) and mental states. Across six studies, the evidence for the actor–observer divergence was scattered and inconsistent on the global person-situation level, but consistently found on the differentiated level. Hence, even though the pervasiveness of the actor–observer asymmetry has been put into question on the traditional global level, it seems to exist on more differentiated levels. Analyzing explanations on such a differentiated level helps to specify the exact nature of the phenomenon. The question arises which level best reflects how people explain human behavior. Interestingly, Malle et al. (2007) did not find consistent evidence for the actor–observer difference when they looked at a very specific level, namely, when they investigated whether observers give more trait explanations than actors. Traits are but one type of stable disposition; possibly, this level was too differentiated to capture the phenomenon.

For the positivity bias, the results of the present study indicate that negative behaviors particularly evoke explanations that refer to uncontrollable events and temporary states. Thus, controllability and stability of the attributed cause need to be considered in addition to its locus when investigating the positivity bias.

Several links assumed in the CEN model, however, did not show in the data of Experiment 1. For example, most of the assumed causes of outcomes were not given as explanations (i.e., temporary states such as nervousness, dispositions such as ability, stimulus attributes such as task difficulty, and events such as luck). This does not necessarily mean that these explanation types would not be regarded as proper explanations for outcomes by participants. Note that they were restricted to mentioning only one explanation for each behavior. They might have given such explanations had they had the opportunity to give more than one explanation. This issue will be addressed in Experiment 2 where participants were asked to try to find explanations of all types for each behavior.

It turned out that actions were explained not only by goals, but also by temporary states and dispositions. Participants gave causes that are assumed to be directly linked to the behavior category as well as indirect causes that are linked to the behavior via intervening causes. This is in accord with CEN, because the links are conceived as transitive so that distant causes can serve as explanations. An action such as “Matthew carries an old lady’s bag to her home” may be explained by the actions goal (e.g., “Matthew wants to help her”), by a state that may have initiated the goal (e.g., “Matthew felt sorry for the old lady”), or by a personal disposition that predisposed Matthew to experience such states and have such goals (e.g., “Matthew is a helpful person”). All causes that are linked to the behavior are assumed to serve as potential explanations. Presumably, a person who names a remote cause as an explanation implies the intervening steps. For instance, we would assume that a person who explains Matthew’s carrying the bag with his dispositional helpfulness implies that his general helpfulness made him want to help the old lady in that particular situation.

The effects of such causal chains and their lengths on the process of generating explanations will be investigated in Experiment 2: Are causes that are directly linked to the behavior generated more easily and faster than remote causes?

## EXPERIMENT 2: RESPONSE LATENCY FOR GENERATING EXPLANATIONS

The CEN model specifies causal paths of varying lengths between two categories. For example, there is an immediate connection with path length 1 from goal to action, from state to action path length is 2 (via goals), and from disposition to action path length is 3 (via states and goals). Three is the maximum path length in the model. There are also category pairs with no direct or indirect connection. For example, there is no causal link going from action to event. That is, an action–explanation should not be appropriate in lay causal thinking if the behavior in question is an event.

If the causal search proceeds along the postulated paths, the ease of generating an explanation of a particular type should decrease with the length of the path that links that explanation type to the behavior type. If there is no path at all between the required explanation and the behavior, it should be very difficult or impossible to generate such an explanation, because it contradicts the lay theory of behavior. In order to test this assumption, the response latency was measured for people to generate an explanation of a particular type for a specific behavior.

Participants were required to produce a certain type of explanation for a behavior item that was presented on a computer screen. As in Experiment 1, each behavior item corresponded to one of the categories. Then, a signal indicated which of the seven categories should be generated as an explanation. Participants had 10 s to generate an explanation of the required type. We measured whether an explanation could be given within these 10 s, and if so, the response latency for generating the explanation. The number of generated explanations was expected to decrease and the response latency to increase with increasing path length; response latency should be particularly long and explanations especially few if there is no link between behavior and explanation according to CEN.

### METHOD

#### Participants

Twenty-two undergraduate psychology students from the same population as in Experiment 1 volunteered to participate. They received course credit for their participation.

#### Behavior descriptions

Eighteen behavior descriptions were presented that were in part taken from Experiment 1. Three behavior descriptions were presented for each of the following categories: goal (e.g., “Cloe wants to change her profession.”), action (e.g., “Sheila invites her neighbors for dinner.”), outcome (e.g., “Monica passes the intermediate exam”), event (e.g., “Mary receives an inheritance”), temporary state (e.g., “Becky is pleased with her birthday gift”), and disposition (e.g., “Tom is an honest person”). In contrast to Experiment 1, goals and dispositions were also included as behaviors because they constitute important comparison cases. According to CEN, participants should find generating explanations easy for goals but difficult for dispositions. All items were formulated in the third person and described positive behaviors.

#### Design and procedure

The independent variable behavior type was manipulated with six levels (goal, action, outcome, event, state, disposition). Each behavior description was presented seven times, once in combination with each of the explanation types. Explanation type thus constituted the second independent variable (with seven levels: goal, action, outcome, event, state, disposition, stimulus attribute). Participants were asked to provide an explanation of the behavior that was of the indicated type. Since there was no reference to a stimulus in the dispositional behavior descriptions (e.g., “Tom is an honest person”), the explanation type *stimulus attribute* was omitted for these behavior items. Each participant performed 123 trials, the order of which was randomized for each participant.

The experiment was run in individual sessions. Before working on the explanation generation task, participants performed a training phase in which they learned the explanation types. The explanation types were explained to participants and they learned one-word keywords for them (GOAL, ACTION, OUTCOME, EVENT, STATE, PERSONALITY, ATTRIBUTE)^3^. Then, they were asked to assign example explanations to the explanation types and were corrected by the experimenter. The practice examples were unrelated to the items used in the explanation generation task. After the training phase the explanation generation task began.

The procedure to measure the response latencies for explanation generation was adopted from a study by Sanitioso et al. (1990) who measured response latencies for the activation of auto-biographic memories. This part of the experiment was run on a computer. Each trial began with a behavior description that appeared on the upper part of the computer screen. The participant hit a key on the computer keyboard when he or she had read the behavior description. Then a prompt appeared on the lower part of the computer screen that indicated which of the seven explanation types was required on that trial. The explanation types were prompted by the one-word keywords that the participants had learned. Participants were instructed to find an explanation of the required type as quickly as possible, at the longest within 10 s. Simultaneously with the explanation-type prompt a bar appeared on the screen that indicated the remaining time by decreasing in length as the 10 s passed. When an explanation of the required type had occurred to the participant, he or she hit a key. After hitting the key the participant said the explanation aloud. The explanations were tape recorded. If the participant could not give an explanation, an acoustic signal indicated when the 10 s had elapsed. That was the end of the trial. The participant started the next trial by hitting a key. Participants performed some practice trials before beginning with the actual task. Every 25 trials they were asked if they needed a short break.

Two dependent measures were taken for each trial: Whether the participant gave an explanation within 10 s or not, and if so, the response latency in millisecond (i.e., the time between the onset of the explanation-type prompt and the participant’s key stroke which indicated that the participant had thought of an explanation).

#### Control measures

Participants performed two additional tasks that were designed to measure control variables: the general speed of reaction for hitting a key and the time required to recollect the meaning of the explanation-type prompts. Furthermore, the time needed to read the behavior descriptions was measured. Since none of the control analyses altered the results, they will not be reported.

#### Elimination of invalid trials

After the experiment participants’ recorded explanations were analyzed in order to identify invalid trials, that is, trials on which participants made mistakes. Invalid trials were eliminated before data analysis. Trials were eliminated for the following reasons: (a) the explanation was of a wrong type; (b) the type was correct, but participants gave a consequence rather than an explanation (e.g., a response such as “Warren will start the job” as an explanation of the behavior item “Warren gets the job for which he applied”); (c) for dispositional behaviors, participants sometimes did not give a causal explanation, but a behavioral indicator of the disposition (e.g., an explanation such as “Tom always tells the truth” for the behavior item “Tom is an honest person”); (d) the explanation was a verbatim repetition of the behavior item; (f) accidental key stroke (as indicated by comments made by the participant). Eighty-five percent of the trials (2306 out of 2706) were classified as correct (i.e., participants gave either an explanation of the required type or no explanation).

#### Path length

From the combinations of behavior types and explanation types a repeated measures factor path length was constructed. Each combination between a behavior type and an explanation type corresponds to a particular path length from the explanation to the behavior. The factor path length has four levels: path length 1 (e.g., from goal to action), path length 2 (e.g., from state to action), path length 3 (e.g., from disposition to action), and no-link (e.g., from action to event). For each participant, the mean response latency and the mean number of explanations for each path length were computed by averaging the trials that corresponded to behavior–explanation combinations with the respective path length.

### RESULTS

Both dependent measures were analyzed by means of a one-way repeated measures analysis of variance with the factor path length. The cell means are shown in Table 3.

**TABLE 3:** **Number of explanations and response latencies for varying path lengths (Experiment 2)**.

Measure	No link	Path length 1	Path length 2	Path length 3
Number of explanations^a^				
*M*	0.64	2.05	2.22	2.38
SD	0.35	0.42	0.62	0.62
Response latency (ms)
*M*	6623	5450	5590	5245
SD	1085	1268	1483	1680

^a^Number of explanations may vary from 0 to 3, because three behavior descriptions for each behavior type were presented.

#### Number of explanations

The effect of path length on the mean number of explanations was significant, *F*(3,19) = 164.77, *p* < 0.001, η^2^ = 0.963. As was expected, the number of explanations that could be generated was much smaller for the no-link condition than when a path exists between behavior and explanation. Surprisingly, when a path exists, the number of explanations increased with path length. The increases from path length 1 to path length 2, *F*(1,21) = 3.66, *p* = 0.21, and from path length 2 to path length 3, *F*(1,21) = 2.68, *p* = 0.36, were not significant, but the difference between path lengths 1 and 3 was significant, *F* (1,21) = 7.08, *p* = 0.045 (*p* values after Bonferroni correction for multiple comparisons). The number of explanations had been expected to decrease rather than increase with path length—this point will be taken up in the discussion.

#### Response latency

There was also a strong effect of path length on response latency, *F*(3,19) = 12.11, *p* < 0.001, η^2^ = 0.657. As hypothesized, response latency was much longer if no path exists than when a path links the explanation type to the behavior type. Unexpectedly, the path lengths 1 to 3 did not significantly differ from each other, *F*(2,20) = 1.49, ns; response latency had been predicted to increase with increasing length of existing paths.

### DISCUSSION

The results of Experiment 2 demonstrate that the no-link combinations of behavior and explanation type contradict a lay causal theory of behavior. The number of explanations is much smaller and the response latency much longer in these cases than when a path exists from explanation to behavior. Participants could not easily generate explanation types that are not causally linked to the behavior type in the CEN model. In the very few instances in which they could provide such an explanation, they required much longer time.

Two aspects of the results were unexpected. The generation of explanations had been assumed to become more difficult as path length increases. Consequently, it had been predicted that the number of explanations would decrease and response latency would increase with path length. However, all explanation types that are linked to the behavior can be generated equally fast, independently of the path length of the connection. And the number of explanations even increases with path length, so that it almost seems as if it becomes easier, not more difficult, to generate an explanation when path length increases.

A plausible interpretation of these findings is that the generation of an explanation is the result of two different processes. One process is the activation of causal hypotheses for a given behavior. This can be thought of as a process of spreading activation (Anderson, 1983b) that starts from the encoded behavior and proceeds along the paths of the network. Apparently, this process is very fast, so that all causes that are linked to the behavior are activated more or less simultaneously—or the differences are so small that our procedure did not detect them. Malle and Holbrook (2012) conducted a study that can be interpreted in terms of the CEN model and whose results suggest that the activation takes longer with increasing path length. Using a similar procedure to Experiment 2, these authors studied the speed and the likelihood with which four types of inferences are drawn from verbal or visual behavior descriptions: the behaviors intentionality, the actor’s desires (i.e., goals), the actor’s beliefs (e.g., what the actor was thinking in the situation), and the actor’s personality traits. They found that intentionality inferences and desire inferences were fastest, belief inferences were slower, and personality inferences were slowest. These types of inferences roughly correspond to the CEN categories goal (intentionality, desire), state (belief), and disposition (personality), which in the CEN model are causally linked to actions with path lengths 1, 2, and 3, respectively. Note that Malle and Holbrook (2012) measured the time it takes to draw an inference of the respective type, which is a more basic process than generating a causal explanation of the type. This is indicated by the average response latencies, which are much shorter in their study than in ours. Possibly, Malle and Holbrook’s (2012) procedure measured the activation process more directly so that increases in response latencies with longer path length showed up.

The second process may be the selection of a cause as an explanation. Presumably, this selection process is what the number of generated explanations measured. Our data suggest, then, that a cause is more easily selected as an explanation if it is an indirect rather than a direct cause of the behavior. The selection process may be guided by conversational principles, apart from the plausibility of the cause. Even if people think that a cause is a plausible one, they may not be willing to explicitly tell it; because they find it too self-evident to be mentioned. Grice (1975) argues that people follow conversational maxims. For example, they want to be informative. That is, they do not want to tell something that they believe the listener already knows. Some authors have applied this reasoning to attributions and explanations (e.g., Hilton, 1990, 2007; Slugoski et al., 1993; Hilton and Slugoski, 2001) and maintain that people will not mention explanations (for example in a questionnaire when participating in a psychological experiment) that they consider as already known or self-evident. Maybe direct causes are too easily inferred and therefore appear self-evident. For example, a goal may be an obvious cause for an intentional action (Malle, 1999). The more interesting cause that is worthwhile mentioning may be the condition that initiated the goal, such as a temporary state or a personal disposition. Self-evidence of a cause may decrease with the length of the causal chain that links the cause to the behavior. That would lead to a preference for selecting indirect causes compared to direct causes.

## GENERAL DISCUSSION

A model called CEN is proposed that incorporates the knowledge-based and hypothesis-driven aspects in the process of causal explanation. The results of two experiments support the basic assumptions of this model. People start an explanation problem with hypotheses about potential causes. These potential causes are derived from the lay theory of behavior; they depend on the type of the behavior that is to be explained. The CEN model specifies the lay theory of social behavior. It consists of two parts: a cognitive taxonomy and inference rules that link the categories of this taxonomy. The taxonomy distinguishes seven cognitive categories that are assumed to be used for both behavior encoding and explanation generation: goals, intentional actions, action outcomes, temporary states, dispositions, uncontrollable events, and stimulus attributes. The inference rules are assumed to reflect causal relations between these categories. Experiment 1 demonstrated that perceived behaviors are cognitively encoded according to the categories, and most explanations generated in free-response format corresponded to one of the categories. Hence, the seven categories postulated in the CEN model seem to reflect the cognitive concepts that make up the lay theory of behavior. Furthermore, two classical attribution tendencies, actor–observer differences and positivity bias, were replicated in Experiment 1, which further corroborates the validity of the categories as explanation types. Furthermore, most of the inference rules were supported in Experiment 1 where free-response explanations were obtained.

Experiment 2 demonstrated that causes that are assumed to be linked to the behavior type are generated faster and more frequently as explanations than causes that are assumed not to be linked to the respective behavior category. This suggests that combinations between a behavior and explanation type that are unrelated in the CEN model are not compatible with the lay causal theory of behavior.

Experiment 2 additionally showed the effects of the path length of the connection between explanation and behavior. All linked explanations were generated equally fast, irrespective of the path length, but the number of explanations increased with path length. This result suggests that all linked causes are activated as causal hypotheses, and that an explanation is selected from this set of hypotheses. A criterion for selecting a cause as an explanation may be found in conversational principles such as the desire to be informative and not to tell anything obvious (Hilton, 1990, 2007). An explanation may be more interesting and worthwhile telling when it refers to a remote, indirect cause rather than to a direct one.

Approaches that deal with lay theories of causal attributions have often been seen as competing with covariational attribution models (Lalljee and Abelson, 1983; Ahn et al., 1995; Ahn and Kalish, 2000; Malle, 2011). However, it seems that these two approaches describe two complementary parts of the attribution process and may thus be integrated (Sutton and McClure, 2001; Rose et al., 2011). Lay theoretical approaches focus upon people’s prior knowledge whereas covariation models focus upon the processing of covariational information. The attribution process always consists of both deductive and inductive components (Young, 1995). Their relative importance may vary depending on the amount of prior knowledge and available information. If we learn that somebody fails an exam and have a strong preconception that this person is lazy, we may not seek any further information in order to explain the failure. On the other hand, if we receive compelling covariation information (e.g., that everybody else also failed), this information may override our preconceptions. Furthermore, in the absence of any beliefs about a plausible cause, people will not be able to infer causation even when they possess covariation information. Lay theories provide causal hypotheses and covariation information may serve to select one of the potential explanations for a behavior. Rose et al. (2011) showed that covariation information is used more if it is informative for testing hypotheses about concrete causal explanations. Sutton and McClure (2001) demonstrated the interplay of lay causal conceptions and covariation information when intentional actions are explained. The covariation principle can be seen as another selection principle besides conversational rules.

In sum, the present studies suggest a two-stage process of attribution: The activation of causal hypotheses and the selection of one or more of the causes as an explanation. The CEN model specifies which hypotheses about potential causes will be activated depending on the type of behavior that is to be explained. The model describes the activation part of the process, or, in Trope and Higgins (1993) terms, the *what* (i.e., the content) of causal attribution. The selection part refers to the cognitive processes that lead to a causal judgment, that is, to the *how* of causal attribution (Trope and Higgins, 1993). The selection process operates on the set of potential causes that have been activated. Numerous attribution principles have been proposed in the literature that are candidates for selection principles. Two broad classes seem to be especially important: Those that are based on additional information, most notably the covariation principle, and those that are based on social processes such as conversational maxims.

The relationship between behavior type and explanation type can be bidirectional. On the one hand, the behavior type determines which explanation types will be considered. For example, if Sally hits Bill, this will appear as an intentional action and a plausible explanation is that Sally was angry with Bill. On the other hand, the explanation for a behavior determines its meaning. If we learn, for example, that Sally stumbled and accidentally hit Bill, this explanation re-categorizes the behavior as an unintentional event. Hence, the behavior type affects the explanation type and the explanation type signals a behavior type. This assumption is supported by Malle (1999). He distinguished reason explanations (which mostly correspond to goal explanations in the present framework) and cause (i.e., non-reason causal) explanations. He found that intentional behavior evokes reason explanations and unintentional behavior cause explanations, and also, that the same behavior seems intentional when explained by a reason but unintentional when explained by a cause.

People may engage in varying amounts of information processing when explaining behavior. They begin the causal search by encoding the behavior as a certain type of behavior, which activates hypotheses about potential causes. These hypotheses may serve as explanations and the process may stop at this point. If they are motivated to do so and have the opportunity, they may search for further information. The present model assumes that people start with searching information that allows them to test their causal hypotheses. If none of these hypotheses is supported by the information, they may try to re-categorize the behavior and test the new hypotheses that arise from the new behavior type (Abelson and Lalljee, 1988).

Most attribution models consider the abstract attribution categories person, stimulus, and situation that have been introduced by Kelley (1967, 1973). Some authors have argued that lay explanations are more concrete and specific than these broad categories (Lalljee and Abelson, 1983; Leddo et al., 1984; Read, 1987; Hilton and Knibbs, 1988; Malle, 1999, 2011; Malle et al., 2000; Kammrath et al., 2005). For instance, “Jack didn’t study” and “Jack is unintelligent” are both person attributions for a failure in an exam, but with different inferential implications, so that they will presumably be distinguished in lay attributional thinking. However, it is an unresolved question which differentiations within the person, stimulus, and situation should be made.

The CEN model offers such a division into more concrete categories. An interesting question is whether there is a preferred level of abstraction at which people generate explanations (Keil, 2006). Such *basic levels* (Rosch, 1978) have originally been proposed for object categories, but have also been identified for cognitive structures in social domains, such as everyday activities (Rifkin, 1985), personality traits (John et al., 1991), and emotions (Shaver et al., 1987). The basic level is generally assumed to be at that level of abstraction where objects are categorized in such a way that objects that are homogeneous with respect to relevant features are grouped together in one category, whereas different categories are heterogeneous with respect to such features. The relevant features of explanations may be assumed to be the traditional attribution dimensions such as intentionality, locus, stability, and controllability. The CEN categories are supposed to bundle causes that are homogeneous with regard to these dimensions and are thus equivalent as explanations. Hence, they may be candidates for a basic level of attribution. Causal thinking should then proceed on the level of concrete actions, states, dispositions, and so on. An experiment that hints in that direction was performed by Smith and Miller (1983). They found that judgments of intention and of the actor’s traits were made faster than causal ascriptions to the person or situation. Presumably, inferences with respect to the actor’s intention and traits were made spontaneously, with the assignment of the inferred cause to the abstract categories of person and situation requiring additional time. In a similar vein, Reeder et al. (2004) found that perceivers spontaneously ascribed specific motives to a target person and that such specific motives were more influential on dispositional inferences than global attributions to the person or situation. Hence, spontaneous causal inferences seem to take place on a level that is more concrete than the traditional tripartite classification of person, stimulus, and situation attributions; the CEN categories aim to capture this preferred level.

In sum, the proposed causal explanation model constitutes a return to Heider’s (1958) original aim of analyzing lay causal theories that people use when explaining behavior. It provides a valuable complement to traditional attribution models such as the covariation model by adding the what to the how of social attribution. The model specifies the content of causal attributions and describes how people understand their own and others’ behavior, which shapes social perception and social interaction and is thus one of the most fundamental aspects of social functioning.

### Conflict of Interest Statement

The authors declare that the research was conducted in the absence of any commercial or financial relationships that could be construed as a potential conflict of interest.

## Footnotes

1 The original stimulus material was in German, as were the first names used in the behavior descriptions. Ten male and 10 female common first names were used that were randomly assigned to the 20 behavior descriptions. When translating the behavior descriptions into English, we decided to also use English first names; they are as close to the German originals as possible.

2 Note that correspondence analysis analyzes row and column *profiles* of a contingency table, rather than absolute frequencies. The distribution of the explanations for the behavior type *action* illustrates this point. In absolute numbers, goals are not the most frequent explanation type given for actions; temporary states, for instance, are more frequent than goals. However, state explanations are more frequent than goal explanations for all behavior types. When looking at the distribution of goal explanations across behavior types, actions are the behavior type that elicits by far the most goal explanations. That is why goal explanations are located closer to action behaviors than state explanations in the correspondence analysis configuration. We believe that this feature of correspondence analysis quite adequately represents the structure of this contingency table. Goal explanations may not be the most frequent type of explanation for actions in absolute terms, but they are the most typical explanation type for actions.

3 The original German keywords were: ZIEL, HANDLUNG, HANDLUNGSERGEBNIS, EREIGNIS, ZUSTAND, PERSÖNLICHKEIT, MERKMAL. We chose “Persönlichkeit” (personality) for disposition in order to select words that are part of common everyday language.
